# Analytical performances of a glycated albumin assay that is traceable to standard reference materials and reference range determination

**DOI:** 10.1002/jcla.24509

**Published:** 2022-05-20

**Authors:** Xinran Tao, Ryosuke Koguma, Yoko Nagai, Takuji Kohzuma

**Affiliations:** ^1^ Diagnostics Department Asahi Kasei Pharma Corporation, IVD Kit Product Group Yurakucho Japan

**Keywords:** analytical performance, diabetes mellitus, glycated albumin, observed value, reference range, standard reference materials

## Abstract

**Background:**

Glycated albumin (GA) is an intermediate‐term marker for monitoring glycemic control (preceding 2–3 weeks) in patients with diabetes mellitus. We evaluated the performance of Lucica Glycated Albumin‐L, a new GA assay that is traceable to standard reference materials and determined the reference range in healthy subjects without diabetes.

**Methods:**

The performance and reference range studies were conducted in accordance with *Clinical and Laboratory Standards Institute (CLSI) Guidelines*. The traceability was established using reference material recommended by the Japan Society of Clinical Chemistry (JSCC).

**Results:**

The coefficient of variation (CV) of overall repeatability, within‐laboratory precision, and overall reproducibility values of GA values were not more than 2.6%, 3.3%, and 1.6%, respectively, among laboratories. The GA values showed good linearity from 173 to 979 mmol/mol (9.4%–54.9%) across the assay range. The GA reference range in 262 healthy subjects was between 183 and 259 mmol/mol (9.9%–14.2%) while that of subjects with diabetes was 217–585 mmol/mol (11.8–32.6%). The reagent was stable for 2 months on the bench at room temperature. The limits of blank, detection, and qualification were 6.9, 7.9, and 9.7 μmol/L for GA concentration, and 3.8, 7.0, and 21.8 μmol/L for albumin concentration, respectively. Hemoglobin slightly affected the assay, while other classical interfering substances had no significant impact.

**Conclusions:**

The present GA assay shows comparable performance to current clinical assays and could be used for intermediate‐term monitoring of glycemic control in diabetes patients.

## Graphical abstract

The Lucica Glycated Albumin‐L assay, which is a new Glycated Albumin assay that is traceable to standard reference materials, shows excellent performance and is comparable to existing assay. The reference range of GA in healthy subjects without diabetes was 183–259 mmol/mol (9.9%–14.2%).

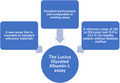

## 1 INTRODUCTION

Diabetes is a complex, chronic illness requiring continuous medical care with multifactorial risk‐reduction strategies beyond glycemic control.^1^ Large‐scale clinical studies such as the Diabetes Control and Complications Trial Research (DCCT),^2^ the Kumamoto Study,^3^ and the U.K. Prospective Diabetes Study (UKPDS)^4^ showed that improving glycemic control inhibits the occurrence and progression of diabetic complications. Hemoglobin A1c (HbA1c) reflects the concentration of mean plasma glucose (MPG) over the last 2–3 months and has been the gold standard for monitoring glycemic control of diabetic patients in clinical practice.^1^ However, HbA1c levels may be inaccurate for hemoglobin variants and abnormal hemoglobin metabolism including anemia, decreased renal function, and gestational diabetes.^5^

Glycated Albumin (GA) is an intermediate‐term glycemic indicator that reflects the glycemic control status for the previous 2–3 weeks due to the albumin half‐life. GA is an important HbA1c substitute for assaying samples with hemoglobin variants and abnormal hemoglobin metabolism because it is unrelated to this pathway. More importantly, GA changes quicker than HbA1c and might be a useful marker for detecting short‐term changes of glycemic control during treatment.^6^, ^7^, ^8^ GA can be used alone or in combination with other biomarkers and may be considered for evaluating glycemic status in patients with advanced chronic kidney disease (CKD) and severe anemia.^9^ HbA1c and GA have similar associations with retinopathy and nephropathy and the associations are strengthened when both measures are considered together in a case study from the Diabetes Control and Complications Trial, and the Epidemiology of Diabetes Interventions and Complications (DCCT/EDIC).^10^ GA is associated with vascular outcomes and mortality from the Atherosclerosis Risk in Communities (ARIC) study, which followed nearly 11,104 patients over 20 years.^11^ There are also similar reports from Chinese and Japanese groups that shows GA is closely related to the onset and progression of diabetic complications in Asian population.^12^, ^13^, ^14^ An Italian group summarized recent updates and advantages of GA as a biomarker for predicting and stratifying the cardiovascular risk.^15^

Historically, GA levels are determined using several methods including boronate affinity chromatography,^16^, ^17^ ion‐exchange chromatography,^18^, ^19^ thiobarbituric acid (TBA) assay,^18^ immunoassay,^20^, ^21^ enzyme‐linked boronate immunoassay,^22^ and high‐performance liquid chromatography (HPLC).^23^, ^24^ In 2002, an enzymatic method for GA measurement was developed by Kohzuma et al, and the improved method in 2004 is traceable using the HPLC method and widely used in clinical practice.^25^, ^26^ However, the target molecules and reference GA ranges differ for each method due to the definition of GA levels.^27^ The Committee on Diabetes Mellitus Indices and the Japan Society of Clinical Chemistry (JSCC) recommended a reference procedure based on isotope dilution mass spectrometry (IDMS) for measuring GA and distributed reference materials for GA determination to standardize GA measurements.^28^

In this study, we evaluated the performance of an enzymatic GA assay that is traceable to the JSCC‐recommended standard reference materials. We compared the standardized new GA assay with the old assay and developed the GA reference range in healthy subjects without diabetes in the United States.

## 2 MATERIALS AND METHODS

### 2.1 Study design and subjects

#### 2.1.1 Reference range study

The GA reference range in the healthy, nondiabetic population in the United States was confirmed following *CLSI Guideline EP28‐A3C*. A single‐visit 2‐site study in the United States was designed. Subjects (≥18 years of age) with good general health based on a medical questionnaire and physician investigator judgment who met the following three conditions were defined as healthy subjects without diabetes and enrolled in the study: (1) HbA1c < 5.7% (38 mmol/mol); (2) fasting glucose <100 mg/dl; and (3) 2‐h plasma glucose in 75 g OGTT (oral glucose tolerance test) <140 mg/dl. Subjects with chronic liver disease or dysfunction, chronic kidney disease, thyroid dysfunction, congestive heart failure, chronic inflammatory diseases, acute inflammatory or infectious disease or disorder within 21 days prior to the study visit were excluded from participation. Similarly, patients who have undergone surgery or inpatient hospitalization within 3 months prior to the study visit were excluded. Furthermore, any medical condition that requires ongoing and chronic treatment with any prescription medication and any other acute or chronic conditions that may significantly influence albumin or glucose metabolism in the opinion of the investigator were excluded. The reference range was constructed based on the 2.5 and 97.5 percentiles for the GA data of healthy subjects. The reference range study was approved by the WCG IRB (20122096). All patients provided informed consent for participation in the study.

#### 2.1.2 Observed value study

The observed value of GA in subjects with diabetes was confirmed following *CLSI Guideline EP28‐A3C*. One hundred and fifty subjects with diabetes (≥18 years of age) from eight different sites were tested. Subjects with end‐stage renal disease, chronic kidney disease of Stage 3 or greater, liver cirrhosis, uncontrolled or untreated thyroid disease, a history within the last 6 months of a blood transfusion, and any other acute or chronic conditions that may significantly influence albumin or glucose metabolism in the opinion of the investigator were excluded from participation. The observed value range was constructed based on the 2.5 and 97.5 percentiles for the GA data of subjects with diabetes. The observed value study was approved by the Tulane University Biomedical IRB (15–765,618). All patients provided informed consent for participation in the study.

#### 2.1.3 Method comparison study

The methods used for comparison studies were the HPLC traceable assay^26^ and the present assay. The present assay under evaluation uses a calibrator that is traceable to standard reference material. The HPLC traceable assay and current assay were used to test 1813 patient samples collected from a multicenter cohort study. The study subjects were identified from the results of a study comparing GA to other glycemic indices.^30^ The correlation between the two assays was studied, and Bland–Altman plots were employed to determine the differences. The method comparison study was approved by The Tulane University Biomedical IRB (15–765,618). All patients provided informed consent for participation in the study.

#### 2.1.4 Assay description and equations

Lucica® Glycated Albumin‐L (Asahi Kasei Pharma) is an enzymatic GA assay that is traceable to the JSCC‐recommended standard reference materials. This assay was used to determine GA expressed in mmol/mol, the ratio of GA concentration to albumin (Alb) concentration using calibrator for Lucica® Glycated Albumin‐L and control for Lucica® Glycated Albumin‐L. The assay was adapted for the Roche Modular P Chemistry Analyzer (Roche diagnostics). Results were automatically reported in mmol/mol. The analyzer calculates the GA value (mmol/mol) using the following calculation formula.

(1) $$\begin{array}{c} \mathrm{GA}\;\left(\text{mmol}/\mathrm{mol}\right)=\mathrm{GA}\;\text{concentration}\;\left(\text{μmol}/L\right)/\text{albumin concentration}\;\left(\text{μmol}/L\right)\times 1000\times 1.012+50.9. \end{array}$$

The conversion formula matching GA (mmol/mol) with the conventional GA (%) is as follows.^29^

(2) $$\mathrm{GA}\;\left(\%\right)=0.05652\times \mathrm{GA}\;\left(\text{mmol}/\mathrm{mol}\right)-0.4217.$$

HbA1c was determined using Tosoh G7/G8 automated analyzer (Tosoh Corporation). Glucose was determined using AU2700 and AU5800 Chemistry analyzers (Beckman Coulter).

All the studies were conducted following the *Clinical and Laboratory Standards Institute (CLSI) guideline* including reference range study, observed value study, and performance study.

### 2.2 Laboratory methods

#### 2.2.1 Precision/Reproducibility

The precision study was performed in accordance with *CLSI Guideline EP5‐A3*. The single‐site precision study tested five serum pools at 2 runs/day in duplicates for 20 days (*N* = 80). The multi‐site precision study tested three serum pools at 5 replicates/run, 1 run/day, for five testing days (*N* = 25) at 3 different laboratories. The time interval between measurements was set at ≥2 h when the precision test measurement was conducted twice a day. The repeatability and reproducibility were calculated as SD and CV%.

#### 2.2.2 Linearity

The linearity study was performed in accordance with *CLSI Guideline EP6‐A* with 11 concentration levels prepared by mixing low and high GA value serum pools. Recovery (%) of the measured samples was calculated, and the polynomial evaluation of linearity was performed. The allowable acceptance criterion was ±5%.

#### 2.2.3 Traceability

The traceability system was established in accordance with “The Committee on Diabetes Mellitus Indices of the Japan Society of Clinical Chemistry‐recommended reference measurement procedure and reference materials for glycated albumin determination.^28^” The traceability and uncertainty of Calibrator for Lucica® Glycated Albumin‐L was studied using the JSCC‐recommended reference material (Glycated Albumin Certified Material, JCCRM 611–1, M, H, HH: ReCCS).

#### 2.2.4 Stability

The stability test was performed in accordance with *CLSI Guideline EP25‐A*. The fluctuations in the measurements were determined, and the allowable drift acceptance criterion was ±10%.

#### 2.2.5 Limit of blank, limit of detection, and limit of quantification

Limit of Blank (LoB), Limit of Detection (LoD), and Limit of Quantitation (LoQ) studies were performed in accordance with *CLSI Guideline EP17‐A2*. Five pooled serums (each pooled serum is prepared using different serums) were treated with resin (Blue Sepharose 6 Fast Flow; GE Healthcare) to remove albumin (ALB) analyte to prepare blank samples for the LoB study. A scalar dilution with saline was used to prepare samples for LoD and LoQ studies.

#### 2.2.6 Analytical interference

The interference study was performed in accordance with *CLSI Guideline EP7‐A2*. Unconjugated bilirubin, conjugated bilirubin, hemoglobin (Sysmex Corporation), glucose, ascorbic acid, triglycerides, uric acid, glibenclamide, metformin hydrochloride, acetylsalicylic acid (FUJIFILM Wako Pure Chemical Corporation, Osaka, Japan), acetaminophen (Toronto Research Chemicals), ibuprofen (ChromaDEX Inc.), hydroxyzine dihydrochloride (LKT Laboratories Inc.), pravastatin sodium (Tokyo Chemical Industry), and penicillin G potassium (Meiji Seika Pharma) were tested. GA value, GA concentrations, and ALB concentrations were measured at basal and increased concentrations of interfering substances. A bias of above 10% was considered a significant interference.

#### 2.2.7 Calculation formula simplification

Equation 1 reports GA (mmol/mol); however, doctors prefer GA (%) in clinical practice. Therefore, Equations 1 and 2 were combined:

(3) $$\begin{array}{c} \mathrm{GA}\;(\%)=0.05652\times (\mathrm{GA}\;\text{conc}.(\text{μmol}/L)/\mathrm{ALB}\;\text{conc}.(\text{μmol}/L)\times 1000\times 1.012+50.9)-0.4217 \end{array}$$

Equation 3 was simplified to obtain Equation 4:

(4) $$\mathrm{GA}\;\left(\%\right)=57.2\times \mathrm{GA}\;\text{conc}.\left(\text{μmol}/L\right)/\mathrm{ALB}\;\text{conc}.\left(\text{μmol}/L\right)+2.46$$

### 2.3 Statistical analysis

The statistical analyses of reference range and observed value study were performed using SAS® version 9.3 or higher (SAS Institute). The method comparison study was studied using the Passing–Bablok method (Starflex). The statistical software Analyse‐it v4 (Microsoft) was used for the analysis of performance studies.

## 3 RESULTS

GA (%) values from 118 samples had a range of 164–1046 mmol/mol (8.9%–58.7%) using Equation 4, which validated the simplification of the equation and was used for subsequent analysis.

### 3.1 Reference range study

Two hundred and sixty‐two healthy subjects without diabetes were included in the reference range study, and the GA reference range was 183–259 mmol/mol (9.9%–14.2%) for the study population of nondiabetic healthy subjects. The reference study included 172 Caucasian subjects, 43 African American subjects, and 41 Asian subjects, which reflect the diversity of the U.S. population. No significant difference was observed between race, sex, age, and ethnicity subgroups (Table 1).

**TABLE 1:** GA reference range of healthy subjects

Subject	Number	HbA1c (%)	HbA1c (mmol/mol)	GA (mmol/mol)	GA (%)
Healthy	262	4.5–5.6	25.7–37.7	183–259	9.9–14.2
Race					
African American	43	4.5–5.6	25.7–37.7	195–269	10.6–14.8
Asian	41	4.8–5.5	28.9–36.6	203–256	11.1–14.0
Caucasian	172	4.5–5.6	25.7–37.7	182–259	9.9–14.2
Sex					
Female	126	4.6–5.6	26.8–37.7	192–260	10.4–14.3
Male	136	4.5–5.6	25.7–37.7	182–256	9.9–14.0
Age					
18–30	123	4.6–5.6	26.8–37.7	186–259	10.1–14.2
31–50	104	4.5–5.6	25.7–37.7	183–258	9.9–14.2
51+	51	5.0–5.6	31.1–37.7	180–260	9.8–14.3
Ethnicity					
Hispanic or Latino	24	4.5–5.6	25.7–37.7	186–259	10.1–14.2
Non‐Hispanic or non‐Latino	238	4.5–5.6	25.7–37.7	182–260	9.9–14.3

### 3.2 Observed value study

One hundred and fifty subjects (*N* = 73 with type 1 diabetes, and *N* = 77 with type 2 diabetes) were included to check the GA range of diabetic subjects. The overall GA range of subjects was 217–585 mmol/mol (11.8–32.6%) with 73 type 1 diabetes subjects exhibiting 256–585 mmol/mol (14.0–32.6%), and 77 type 2 diabetes subjects showing 214–598 mmol/mol (11.7–33.4%).

Subjects with diabetes were divided into two classes: poorly controlled group 1 consisting of 98 subjects with an HbA1c value within the range of 7.5%–12%, and well‐controlled group 2 consisting of 52 subjects with an HbA1c value <7.5%. A GA range of 292–598 mmol/mol (16.1–33.4%) and 214–386 mmol/mol (11.7–21.4%) was observed for group 1 and group 2, respectively. No significant difference was found between race, sex, age, and ethnicity subgroups (Table 2).

**TABLE 2:** Observed GA values in subjects with diabetes

Subject	Unit	Group 1		Group 2		Total	
Diabetic	mmol/mol	*N* = 98	292–598	*N* = 52	214–386	*N* = 150	217–585
	%		16.1–33.4		11.7–21.4		11.8–32.6
Race^a^							
African American	mmol/mol	*N* = 13	301–598	*N* = 4	267–311	*N* = 17	267–598
	%		16.6–33.4		14.7–17.2		14.7–33.4
Caucasian	mmol/mol	*N* = 80	294–618	*N* = 46	214–386	*N* = 126	217–581
	%		16.2–34.5		11.7–21.4		11.8–32.4
Sex							
Female	mmol/mol	*N* = 53	259–581	*N* = 27	204–387	*N* = 80	214–558
	%		14.2–32.4		11.1–21.5		11.7–31.1
Male	mmol/mol	*N* = 45	301–652	*N* = 25	227–386	*N* = 70	236–652
	%		16.6–36.4		12.4–21.4		12.9–36.4
Age							
18–30	mmol/mol	*N* = 14	333–598	*N* = 7	277–387	*N* = 21	277–598
	%		18.4–33.4		15.2–21.5		15.2–33.4
31–50	mmol/mol	*N* = 26	319–659	*N* = 17	204–374	*N* = 43	215–540
	%		17.6–36.8		11.1–20.7		11.7–30.1
51+	mmol/mol	*N* = 58	259–535	*N* = 28	214–386	*N* = 86	220–517
	%		14.2–29.8		11.7–21.4		12.0–28.8
Diabetes type							
Type 1	mmol/mol	*N* = 47	298–585	*N* = 26	246–387	*N* = 73	256–585
	%		16.4–32.6		13.5–21.5		14.0–32.6
Type 2	mmol/mol	*N* = 51	259–598	*N* = 26	204–323	*N* = 77	214–598
	%		14.2–33.4		11.1–17.8		11.7–33.4
Ethnicity							
Hispanic or Latino	mmol/mol	*N* = 22	259–659	*N* = 7	236–334	*N* = 29	236–659
	%		14.2–36.8		12.9–18.5		12.9–36.8
Non‐Hispanic or non‐Latino	mmol/mol	*N* = 76	292–585	*N* = 45	214–386	*N* = 121	217–539
	%		16.1–32.6		11.7–21.4		11.8–30.0

^a^ Other races were omitted in this range analysis due to the limited sample size.

### 3.3 Method comparison study

**FIGURE 1:**
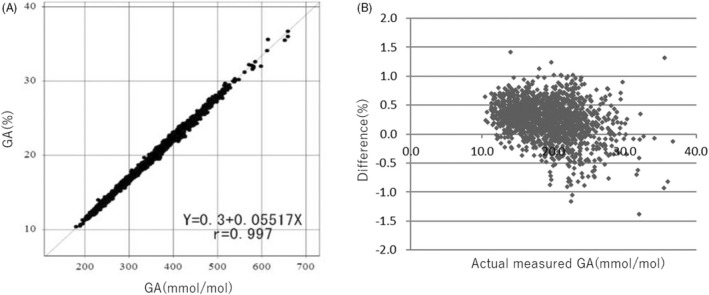
Method comparison study. The Calibrator and Control were traceable to the JSCC‐recommended GA material JCCRM‐611–1. The traceability system is maintained and prevents drift by comparing secondary calibrator measurements with the calibration values of each product‐related calibrator. (A) Correlation between the HPLC traceable assay (%) and the present assay (mmol/mol). (B) Bland–Altman plots between actual measured GA value (%) and differences (%)

The correlation coefficient was *r* = 0.997 between the HPLC traceable assay (%) and the present standardized GA assay (mmol/mol) (Figure 1) using the following correlation equation: GA (%) = 0.05517 × GA (mmol/mol) + 0.300. Calculated GA (%) was 94.2%–107.9% of the measured GA (%), indicating that there was strong consistency between the unit % and mmol/mol.

### 3.4 Precision/reproducibility

The overall repeatability (% CV) in the single‐site precision study was within 0.4%–3.7% for GA values, GA concentrations, and Alb concentrations, while the overall within‐laboratory precision (% CV) for these values was within 0.8%–4.2% (Table 3a). Meanwhile, the overall reproducibility (% CV) of GA values, GA concentrations, and Alb concentrations in the multi‐site precision study was within 0.9%–1.6%, 1.2%–2.5%, and 1.1%–1.2%, respectively (Table 3b).

**TABLE 3:** Precision/reproducibility study

(a) Single‐site precision study (*N* = 80)			
Sample	Mean	Repeatability (% CV)	Within‐laboratory (% CV)
Precision summary of glycated albumin values (mmol/mol)			
Pool serum 1	185.2	1.7%	2.2%
Pool serum 2	228.0	0.8%	1.1%
Pool serum 3	359.9	0.7%	0.9%
Pool serum 4	877.7	0.8%	0.9%
Pool serum 5	229.6	2.6%	3.3%
Precision summary of glycated albumin concentrations (μmol/L)			
Pool serum 1	76.14	2.2%	2.8%
Pool serum 2	117.55	0.7%	1.4%
Pool serum 3	212.42	0.6%	1.0%
Pool serum 4	516.09	0.5%	0.8%
Pool serum 5	51.60	3.7%	4.2%
Precision summary of albumin concentrations (μmol/L)			
Pool serum 1	573.82	0.6%	1.0%
Pool serum 2	671.91	0.7%	0.8%
Pool serum 3	695.71	0.7%	0.8%
Pool serum 4	631.68	0.6%	0.8%
Pool serum 5	292.20	1.1%	1.2%

(b) Multi‐site precision study (*N* = 75)				
Sample	Mean	Repeatability (% CV)	Within‐laboratory (% CV)	Reproducibility (% CV)
Precision summary of glycated albumin values (mmol/mol)				
Pool serum 1	187.7	0.8%	1.0%	1.6%
Pool serum 3	363.1	0.7%	0.9%	0.9%
Pool serum 4	888.2	0.7%	0.8%	0.9%
Precision summary of glycated albumin concentrations (μmol/L)				
Pool serum 1	78.43	1.0%	1.6%	2.5%
Pool serum 3	217.21	0.8%	1.3%	1.3%
Pool serum 4	528.76	0.8%	1.2%	1.2%
Precision summary of albumin concentrations (μmol/L)				
Pool serum 1	580.40	1.1%	1.2%	1.2%
Pool serum 3	704.10	0.9%	1.1%	1.1%
Pool serum 4	639.09	0.9%	1.1%	1.1%

### 3.5 Linearity

The GA value showed good linearity from 173 to 979 mmol/mol (9.4%–54.9%) across the assay range (*y* = 0.993*x* + 2.880; *R* ^2^ = 0.9998) with a good recovery from 98.9% to 100.9%. The linear GA and Alb concentration ranges were from 52.53 to 605.90 μmol/L (*y* = 0.9932*x* − 0.6216) and 185.40–1196.03 μmol/L (*y* = 0.9983*x* + 4.290), respectively.

### 3.6 Traceability

**FIGURE 2:**
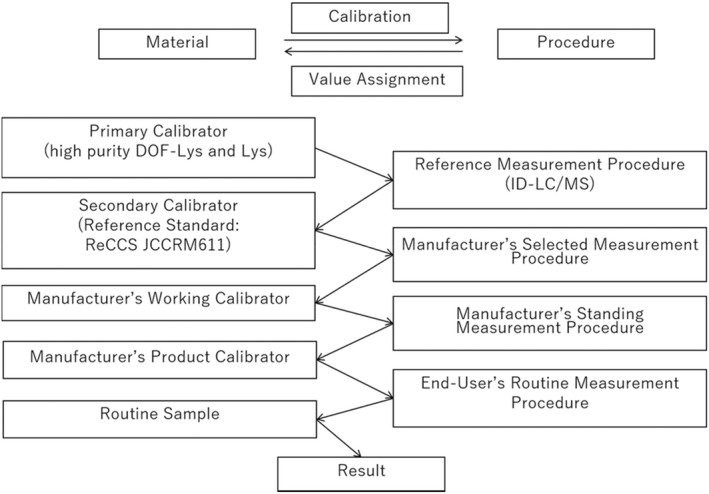
Traceability system for the glycated albumin value (mmol/mol)

The calibrator and control were traceable to the JSCC‐recommended GA material JCCRM‐611 (Figure 2). The traceability system is maintained and prevents drift by comparing JCCRM‐611 measurements with the calibration values of each product‐related calibrator. The ratios of the measured values and the certified values of JCCRM611 were between 101% and 103%. The expanded uncertainty of the manufacturer's product calibrator (Calibrator for Lucica® Glycated Albumin‐L) was 3.217%.

### 3.7 Stability

The shelf‐life for the reagents was 12 months when refrigerated within a temperature range of 2 and 8°C. The reagent was stable for 2 months on the bench at room temperature.

### 3.8 Limit of quantification, limit of blank, and limit of detection

The LoQ, LoB, and LoD for GA and Alb concentrations were 9.7 μmol/L and 21.8, 6.9, and 3.8 μmol/L, and 7.9 and 7.0 μmol/L, respectively.

### 3.9 Analytical interference

Unconjugated bilirubin, conjugated bilirubin, glucose, ascorbic acid, triglycerides, and uric acid showed no significant interference to the present GA assay at the following concentration (accepted bias <±10%): unconjugated bilirubin <20.0 mg/dl, conjugated bilirubin <20.0 mg/dl, glucose <1000 mg/dl, ascorbic acid <100 mg/dl, triglycerides <1516 mg/dl, and uric acid <23.5 mg/dl. Hemoglobin slightly affected the assay only at >288 mg/dl. Hemoglobin at 384 mg/dl decreases the glycated albumin value in serum at 240 mmol/mol (13.1%) by 12.9%, and at 467 mmol/mol (26.0%) by 9.9%.

### 3.10 Calculation formula simplification

The differences between Equations 2 and 4 GA values were within −0.1 to 0.1%. The results obtained by Equation 4 were equivalent with the results of Equation 2.

## 4 DISCUSSION

In this study, a standardized GA assay that is traceable to JSCC‐recommended standard reference materials demonstrated satisfactory performance for precision, linearity, LoB, LoD, LoQ, and analytical interference. The calculation formula to report GA (%) directly to doctors was simplified by measuring 1813 patient samples using the correlation equation: GA (%) = 0.05517 × GA (mmol/mol) + 0.300 (*r* = 0.997). This equation is comparable to a previous unit conversion formula established by Sato et al: GA (%) = 0.05652 × GA (mmol/mol) − 0.4217 (Equation 2, *r* = 0.999).^29^ Our results confirmed that both equations could be used to convert GA values between % and mmol/mol.

A reference range of 183–259 mmol/mol (9.9%–14.2%) was developed in 262 nondiabetic healthy subjects in this work. Several studies have discussed the GA reference range in the U.S. population. For example, the GA reference range was between 11.9% and 15.8% from a study of 201 subjects with differences observed between African American and Caucasian samples.^24^ The GA difference between African American and Caucasian subjects was similar to differences in HbA1c.^31^ The GA reference range was 10.7%–15.1% in a healthy population of 1799 individuals (mean age: 55 years old; 51% female, 15% African American).^32^ Therefore, the reference range in this study is lower compared with previous studies. Previous studies were based on subjects without diabetes, and there is a high possibility that both healthy subjects and prediabetic subjects were included. In contrast, prediabetic subjects, including impaired fasting glucose and impaired glucose tolerance, were excluded from this study. This inclusion/exclusion criteria is the likely main cause of the lower reference range in this work, although the limited sample size might also affect the results.

Although African Americans showed a higher GA reference range (10.6%–14.8%) than Caucasians (9.9%–14.2%) and Asians (11.1%–14.0%), no significant difference was observed in the present study. Selvin et al reported that black people have significantly higher HbA1c and GA levels than white people in a community‐based study.^31^ The GA reference interval of 663 healthy individuals ranged from 10.7% to 15.2% in a study from Cape Town, South Africa, with the observed GA for Black Africans significantly higher than that for Caucasians (*p*‐value of 0.0025).^33^ On the contrary, 1334 Italian blood donors had a GA upper reference limit of 14.5% (95% CI: 14.3%–14.7%) for Caucasians,^34^ which is comparable with this study. The impact of sex is also controversial. Although a Chinese group raised the possibility that sex may influence GA,^35^ no evidence was found to support this conclusion in our study. This discrepancy might be resolved using a systemic and larger meta‐analysis study to determine the reference range of the population since this study had a limited sample size and the definition of the reference group was not identical among different studies.

Subjects with diabetes had a GA range of 217–585 mmol/mol (11.8%–32.6%) with HbA1c values between 5.9% and 11.2%. The GA levels in subjects with type 1 diabetes were higher than that in type 2 diabetes. Furthermore, GA levels in the poorly controlled group were higher than that of the well‐controlled group. There was some overlap in healthy subjects and subjects with diabetes in the well‐controlled group with a stable diabetic management program. A reasonable differential interpretation of GA values could be made in the overlapping region of known nondiabetic, prediabetic, or diabetic subjects. A value in the overlapping region may suggest successful efforts in the preceding weeks to reduce blood glucose levels to the normal or near normal range, especially for a known subject with diabetes. A comparison of GA values from patients with diabetes with the GA reference range of healthy subjects is useful to determine patients' glycemic control status.

However, body mass index (BMI) should be noted because it may exert a negative effect on GA.^36^ Furthermore, changes in albumin metabolism influence GA values (Table 4).^37^ Lower GA values are observed relative to plasma glucose levels in patients with conditions involving increased albumin metabolism: nephrotic syndrome, hyperthyroidism, Cushing's syndrome, and obesity; and higher GA values are observed in relation to glycemia in patients with decreased albumin metabolism: liver cirrhosis and hypothyroidism. The reason for the negative impact of BMI on GA levels^38^ is unclear; however, it may be related to albumin metabolism, catabolism, and inflammation in obese individuals, and other factors. Recent studies have found that GA may underestimate glycemic level in obese subjects. Meanwhile, sex hormone‐binding globulin is positively correlated with serum GA levels, which is independent of total or segmental body fat.^39^

**TABLE 4:** Physiological conditions with aberrant GA values

Conditions with high GA Liver cirrhosis Hypothyroidism
Conditions with low GA Nephrotic syndrome Hyperthyroidism Cushing's syndrome Obesity

To our knowledge, the present GA assay is the first FDA‐approved glycated albumin kit that is traceable to the JSCC‐recommended standard reference materials. This ensures reliable and stable GA values in clinical use resulting in improved reliability compared with fructosamine tests.^30^ GA is significantly correlated with short‐term mean blood glucose and long‐term HbA1c.^30^ Monitoring GA using the present Lucica® Glycated Albumin‐L assay is more useful than fructosamine and other glycemic indices in clinical situations from the United States requiring intermediate‐term monitoring of glycemic control since the analytical performance and reference range of the present JSCC‐traceable GA assay is comparable to the HbA1c gold standard assay for glycemic control markers.^30^ Therefore, utilizing the Lucica® Glycated Albumin‐L assay should provide more information to clinicians leading to better management of patients with diabetes.

In conclusion, the Lucica® Glycated Albumin‐L assay is traceable to standard reference materials, shows excellent performance, and could be a useful diagnostic test for the intermediate‐term monitoring of glycemic control in patients with diabetes.

## AUTHOR CONTRIBUTIONS

X.T and T.K. conceived the reference range study and participated in analysis and interpretation of the data. K.R and Y.N conducted the performance study and participated in analysis and interpretation of the data. All authors participated in the drafting and critical revision of the manuscript. All authors had full access to the data in the study and had final responsibility for the decision to publish.

## CONFLICT OF INTEREST

All authors are employees of Asahi Kasei Pharma.

## PATIENT CONSENT STATEMENT

All patients provided informed consent for participation in the study.

## Data Availability

The dataset generated during the current study is not publicly available but is available from the corresponding author on reasonable request.
